# Dissecting of the Deterioration in Eating Quality for Erect Panicle (Ep) Type High Yield *Japonica* Super Rice in Northest China

**DOI:** 10.1186/s12284-022-00561-9

**Published:** 2022-03-08

**Authors:** Sibo Chen, Shuangjie Chen, Yihui Jiang, Qing Lu, Zhongyuan Liu, Wanying Liu, Xuhong Wang, Wenhua Shi, Quan Xu, Jian Sun, Fan Zhang, Liang Tang

**Affiliations:** 1 Rice Research Institute, Shenyang Agricultural University/Key Laboratory of Northern Japonica Super Rice Breeding, Ministry of Education, Shenyang, 110866 China; 2 Institute of Crop Sciences/National Key Facility for Crop Gene Resources and Genetic Improvement, Chinese Academy of Agricultural Sciences, 12 South Zhong-Guan-Cun Street, Haidian District, Beijing, 100081 China

**Keywords:** Erect panicle, Eating quality, Nitrogen metabolism, Grain filling, Grain protein

## Abstract

Ep type is an important morphological improvement (following dwarf breeding and ideal plant type) that has contributed to breeding super-high yielding, and shows a pleiotropic effect in increasing grain yield and also nitrogen-use efficiency (NUE) in rice. Nevertheless, it remains unclear whether Ep has adverse effects on eating quality and how it affects nitrogen uptake and assimilation. In this study, we developed a pair of near-isogenic lines (NILs) for panicle type (NIL-Ep, NIL-non Ep) in the Liaogeng 5 (LG5) and Akihikari (AKI) backgrounds. Rice plants of the NIL-Ep had higher grain numbers per panicle in the middle to bottom spike positions than plants of the NIL-non Ep. The increased grain number is not only is the key factor leading to increased yield but also is the reason for reduced the eating quality. The content of prolamin and glutelin was significantly higher in NIL-Ep, which resulted in higher hardness and worse viscosity of rice after cooking. In addition, the activity of several essential enzymes catalyzing nitrogen metabolism was higher in the NIL-Ep line grains than in the NIL-non Ep, especially from the mid to late grain filling stage. Based on these results, we conclude that Ep positively regulates grain protein accumulation, primarily through enhancing the activity of enzymes involved in nitrogen assimilation and redistribution during the mid to late grain-filling stage, resulting in excessive accumulation of grain protein and decreased eating quality.

**Supplementary Information:**

The online version contains supplementary material available at 10.1186/s12284-022-00561-9.

## Background

Rice is a dietary staple for more than half of China’s population, and so improving rice production is crucial for ensuring food security (Godfray and Garnett 2014). Over the past half century, rice yields have been dramatically improved through successful breeding and the use of high-yielding varieties (Peng et al. 2008). In particular, the release of a series of super rice varieties has pushed rice production to a new peak in northern China (Tang et al. 2017a). From 1980 to 2019, with the popularization and use of a series of high-yielding Ep varieties led to dramatic increases in both the cultivated area (from 2.8 to 9.8 million hectares) and yield (from 4013.2 to 7429.5 kg/hm^2^) of *japonica* rice. In 2019, northeast China was the dominant region, with cultivated area of 5.3 million hectares, accounting for 53.7% of the total area of *japonica* rice (Tang and Chen 2021).

During the twenty-first century, China’s per capita consumption of *japonica* rice has continued to rise and the average annual consumption increased from 37.8 to 55.4 kg, resulting in a sustained growth in the domestic rice market demand for high-quality *japonica* rice (Tang and Chen 2021). Although the Ep type plays an important role in promoting the yield of northern *japonica* rice, there is room for improvement in the eating quality (EQ) of Chinese rice to match Japanese high-quality rice (Wang et al. 2019). In recent years, breeders have paid increasing attention to the eating quality of rice. Maintaining high yield while improving inferior quality has become the main problems to be solved in this rice-growing region.

The eating quality of rice is determined mainly by its intrinsic components. The starch and protein contents and composition have significant effects on the hardness, elasticity and viscosity of rice (Crofts et al. 2017; Kashiwagi 2021). In the pasting process, protein will affects the swelling and water absorption of starch granules, and so protein content is negatively correlated with EQ (Balindong et al. 2018; Zhu et al. 2020). Our previous studies have investigated the effect of Ep-type on quality, but there is no systematic explanation for EQ because of material limitations (Fei et al. 2019). The main gene controlling panicle type in Ep-type super rice varieties *dense and erect panicle 1 (dep1)* is also a nitrogen-use efficiency gene (Sun et al. 2014). High-efficiency nitrogen utilization plays a very important role in improving rice yield, but there is still no clear explanation for its interaction with EQ. In order to further optimize the balance between yield and quality at a higher level in northern China, the following two important scientific issues need to be further addressed. Whether and how Ep can affect EQ?

## Results

### Multi-year Phenotypic Assessment of Yield and Taste Quality of Different Panicle Types

**Fig. 1:**
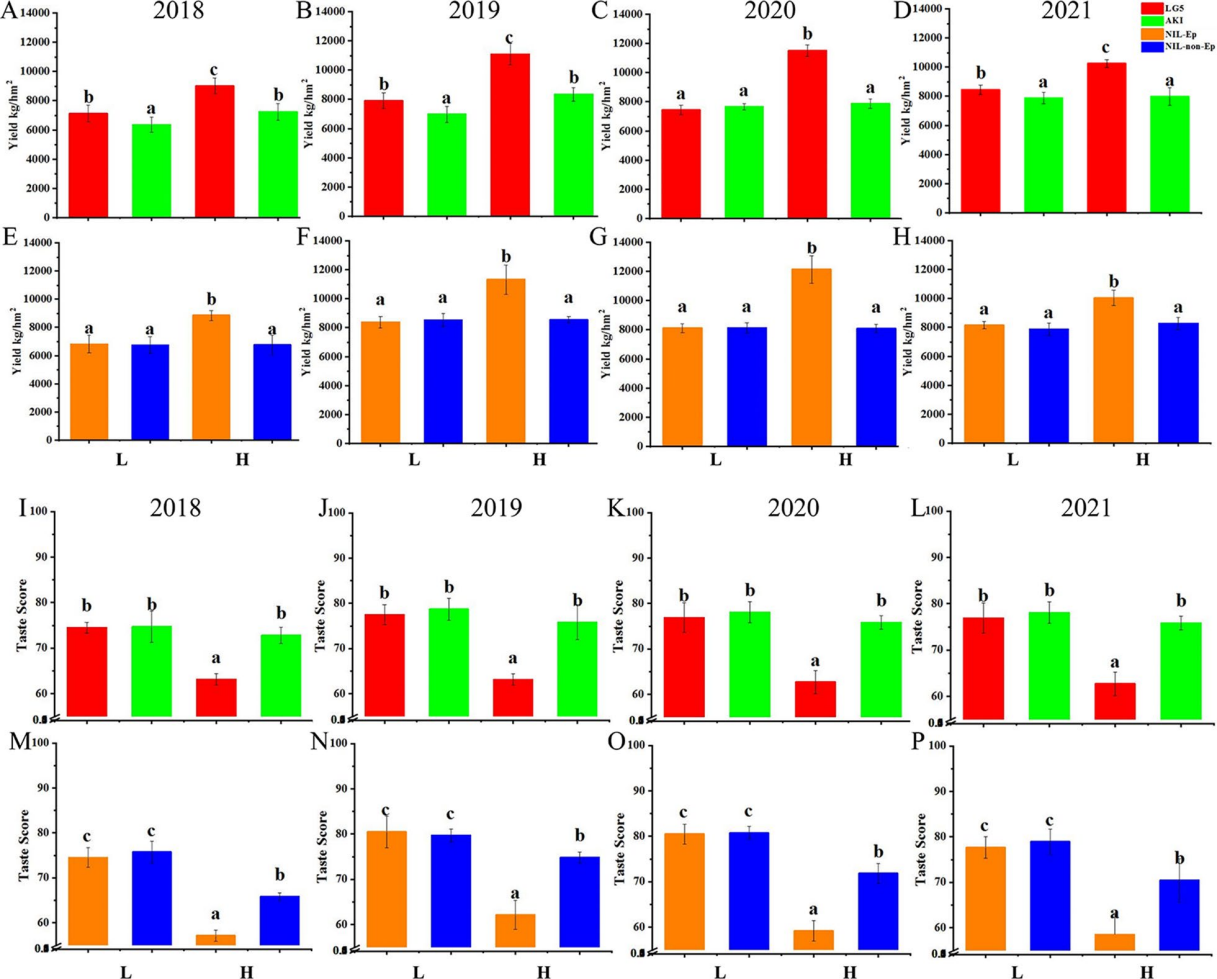
Phenotypes of yield and taste quality of test meterials from 2018 to 2021. **A**–**H** Yield for LG5, AKI, NIL-Ep and NIL-non Ep. **I**–**P** Taste score for LG5, AKI, NIL-Ep and NIL-non Ep. (a), (b) Significance at the 0.05 level. **L**, **H** Low and high nitrogen treatments respectively

**Fig. 2:**
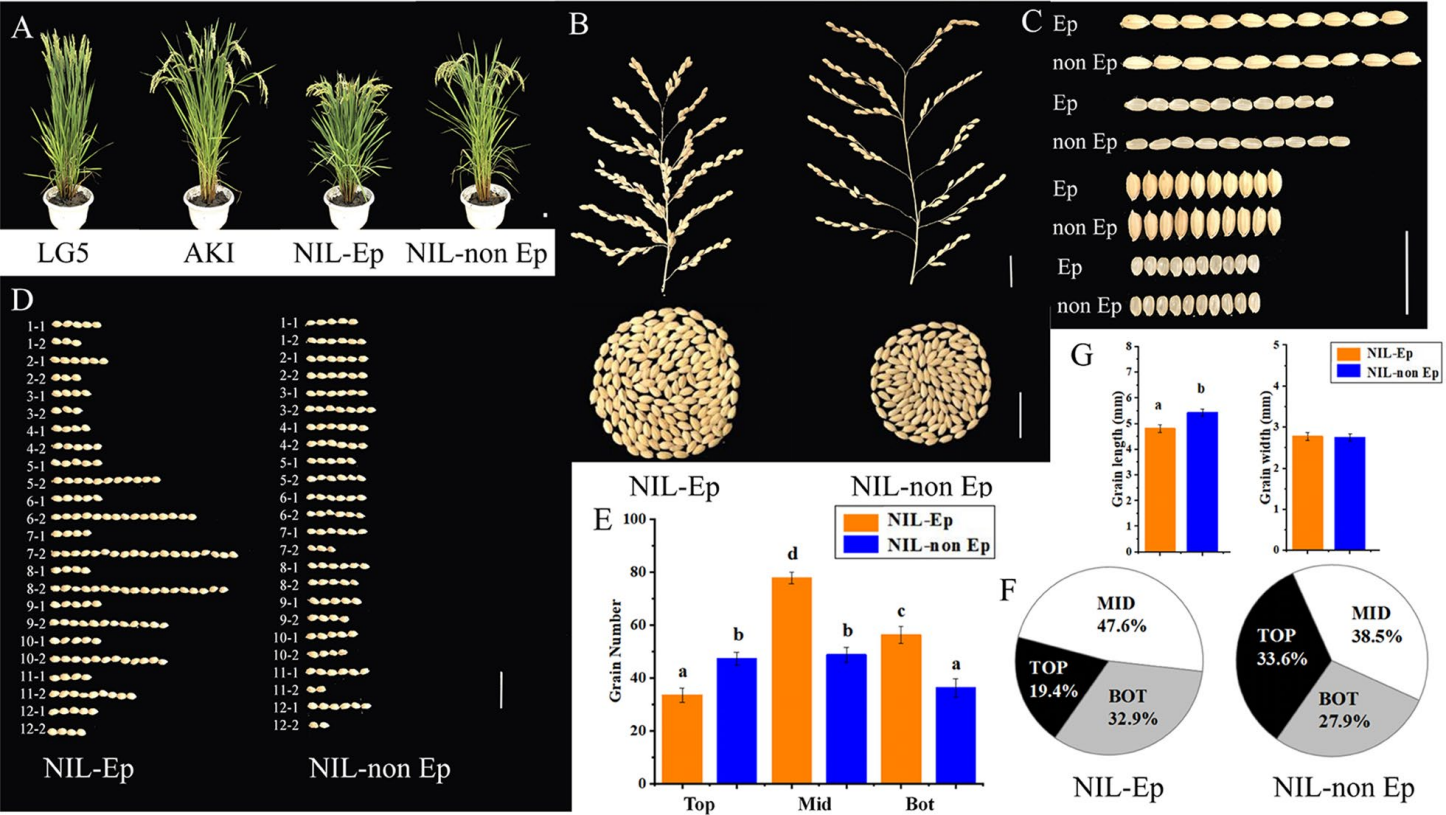
The yield performance and grain shape of NIL-Ep and NIL-non Ep plants. **A** The four experimental materials plant in pot under high nitrogen treatment. **B** The panicles and grain numbers per panicle of the NIL-Ep and NIL non Ep. **C** The grain size of the NIL-Ep and NIL non Ep. **D** The grain numbers of different panicle locations for NIL-Ep and NIL non Ep, divided rice panicles into 24 positions from 1–1 to 12–2 according to the origin positions of branches. e.g. 1–1 represented primary branches at the top and 12–2 represented the secondary branches at the bottom. **E** Difference analysis of grain number in different panicle parts, the panicle is divided into 3 parts namely top (top, from 1–1 to 4–2 panicle positons), middle (mid, from 5–1 to 8–2 panicle positons) and bottom (bot, from 9–1 to 12–2 panicle positons) respectively. **F** Proportion of grain number in different panicle positions. **G** Difference analysis of grain shape. (a), (b) Significance at the 0.05 level. Scale bar, 2 cm

To investigate the effects of Ep on the grain yield and taste quality under different N fertilizer treatments, we used two Nitrogen fertilizer treatments as low (L) and high (H). The results for plants grown under high nitrogen condition are shown in Fig. 2A. The yield and taste quality traits were investigated over four years as shown in Fig. 1. Under L treatment, the LG5 yield was significantly higher than the AKI yield in 2018, 2019, and 2021 (Fig. 1A, B, D) but not significantly different with in 2020 (Fig. 1C). No significant difference was observed between the two NILs in L treatment (Fig. 1E–H). Under H treatment, LG5 yield was significantly higher than AKI in all four years (Fig. 1A–D), and the NILs showed the same pattern as their parents (Fig. 1E–H).

For taste quality, under L treatment, there was no significant difference between LG5 and AKI. Under H treatment, the taste quality of LG5 was significantly lower than that of AKI, and the quality of AKI did not decrease with the higher nitrogen fertilizer (Fig. 1I–L). The taste quality of the NILs showed the same pattern as that of their parents under the same treatment. However, NIL-non Ep showed significantly lower taste quality under H treatment, compared with L treatment (Fig. 1M–P).

### Higher Grain Number per Panicle in Middle and Bottom Parts is the Main Factor Underlying Increased Yield in Ep

There was no significant difference in yield under L treatment, but there was a significant difference in yield and its components between the NILs under H treatment (Table 1). Under the different treatments, the yield and yield component traits of the NILs were consistent with their parents, AKI and LG5. The average panicle number per square metre (PNP) and grain number per panicle (GNP) in NIL-Ep were 19.9% and 29.5% higher, respectively, than in NIL-non Ep (Fig. 2B). In order to clarify the source of differences in GNP, we assigned the rice panicles into 24 positions from 1-1 to 12-2 according to the origin positions of the branches. The results showed that a significant increase in secondary grain number (SGN) from the 5th spike to 12th spike explained the difference in GNP (Fig. 2D). Subsequently the panicle was divided into 3 parts namely top (top) (panicle positions 1-1 to 4-2), middle (mid, panicle positons 5-1 to 8-2) and bottom (bot, panicle positions 9-1 to 12-2). Compared with the NIL-non Ep, grain number was significantly higher in NIL-Ep, mainly in the middle and bottom locations (Fig. 2E). The panicle weight ratio of each part differed, and the panicle weight ratio in the middle to bottom part was 21.5% and 18.7% higher in the Ep line (Fig. 2F). Although Ep showed a lower thousand-grain weight (TWG) because the grain length was significantly lower (Fig. 2C, G), the extremely significantly higher GNP and PNP was the key factor leading to the significantly higher yield; the contribution rates of the three factors were -7.1%, 29.5% and 19.9% respectively.

**Table 1:** Performance of grain yield related traits for LG5, AKI, NIL-Ep, NIL-non Ep

Treatment	Panicle type	HD	PH (cm)	PL (cm)	PNP	GNP	FGN	PBN	PGN	SBN	SGN	TWG (g)	GY (kg/hm^2^)
L	LG5	115	109.82 ± 2.91	14.97 ± 0.63	448.10 ± 32.71	89.50 ± 2.74	76.50 ± 2.74	10.50 ± 1.05	57.67 ± 2.42	11.33 ± 1.21	31.83 ± 2.64	25.21 ± 1.12	7446.67 ± 310.05
	AKI	114	118.77 ± 1.37	17.22 ± 0.86	433.29 ± 36.51	90.83 ± 6.49	78.17 ± 10.76	10.67 ± 0.82	59.33 ± 4.18	11.67 ± 1.75	31.50 ± 4.59	26.53 ± 0.23	7646.67 ± 215.72
	p	n.s	*	*	n.s	n.s	n.s	n.s	n.s	n.s	n.s	*	n.s
	NIL-Ep	110	92.58 ± 0.90	13.74 ± 0.55	474.46 ± 39.23	92.94 ± 5.80	79.94 ± 5.80	10.71 ± 1.21	61.53 ± 6.25	11.53 ± 0.87	31.41 ± 3.55	23.61 ± 0.46	8106.67 ± 292.97
	NIL-non Ep	111	118.29 ± 2.04	16.87 ± 1.01	464.75 ± 28.82	90.50 ± 6.62	79.00 ± 11.45	10.69 ± 1.08	60.25 ± 5.37	11.19 ± 1.64	30.25 ± 4.01	26.32 ± 0.21	8116.67 ± 363.64
	p	n.s	***	**	n.s	n.s	n.s	n.s	n.s	n.s	n.s	**	n.s
H	LG5	118	111.52 ± 1.43	15.82 ± 0.48	497.73 ± 30.14	147.00 ± 6.42	126.00 ± 6.51	12.60 ± 1.02	71.40 ± 3.61	25.40 ± 1.62	75.60 ± 3.26	24.24 ± 0.14	11,523.33 ± 322.94
	AKI	116	127.43 ± 4.27	18.25 ± 0.86	427.41 ± 44.11	120.76 ± 9.32	108.71 ± 12.45	12.00 ± 0.61	68.71 ± 3.85	18.41 ± 2.94	52.06 ± 10.47	25.24 ± 0.37	7870.00 ± 315.75
	p	n.s	***	**	**	***	***	n.s	n.s	***	***	*	***
	NIL-Ep	113	94.24 ± 1.83	16.28 ± 0.45	524.39 ± 25.33	167.60 ± 5.50	141.80 ± 6.26	12.60 ± 0.55	60.60 ± 3.58	29.00 ± 2.00	107.00 ± 4.85	23.22 ± 0.13	12,145.33 ± 947.07
	NIL-non Ep	114	115.22 ± 4.80	19.16 ± 0.96	437.08 ± 24.36	129.47 ± 6.22	114.53 ± 7.62	12.06 ± 0.90	69.41 ± 2.76	19.76 ± 2.49	60.06 ± 5.80	24.99 ± 0.20	8076.67 ± 285.72
	p	n.s	***	**	***	***	***	n.s	**	***	***	**	***

HD, heading date; PH, plant height; PL, panicle length; PNP, panicle number per square meter; GNP, grain number per panicle; FGN, filled grain number per panicle; PBN, primary branches; PGN, primary grain number; SBN, secondary branches number; SGN, secondary grain number; TGW, thousand-grain weight; GY, grain yield

*, **, ***Significance at *p* < .05; *p* < .01; and *p* < .001, respectively

### Eating Quality and Protein Content in Different Panicle Positions

**Fig. 3:**
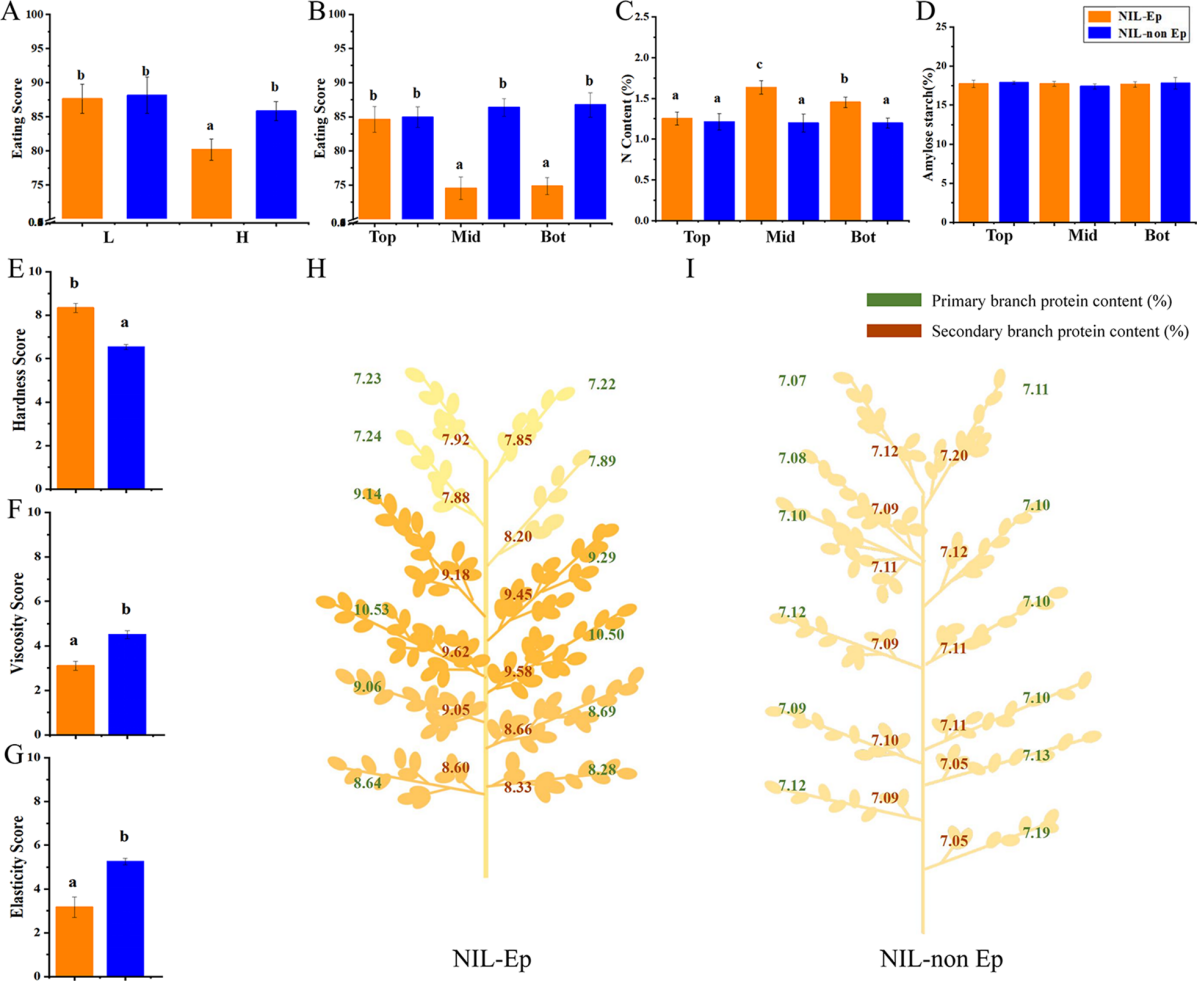
The eating quality performance and grain protein, starch content of NIL-Ep and NIL-non Ep plants. **A** Eating quality under two nitrogen fertilizer treatments. L, H: Low and high nitrogen treatments respectively. **B** Eating quality of different panicle locations under H treatment, the panicle is divided into 3 parts namely top (top, from 1–1 to 4–2 panicle positons), middle (mid, from 5–1 to 8–2 panicle positons) and bottom (bot, from 9–1 to 12–2 panicle positons) respectively. **C** N content of different panicle locations under high nitrogen treatment. **D** Amylose content of different panicle locations under high nitrogen treatment. **E**–**G** Components of eating quality of NIL-Ep and NIL-non Ep under high nitrogen treatment. **H**, **I** Protein content (%) of different panicle locations under high nitrogen treatment. (a), (b) Significance at the 0.05 level

The EQ of rice is a complex sensory trait affected by the hardness, viscosity, elasticity and other indicators of rice. To accurately and objectively measure the EQ of the tested materials, we adopted two sets of evaluation systems: artificial tasting and machine evaluation. The two evaluation systems showed the same results.Under L treatment, there was no significant difference in the EQ between NILs; under H treatment the EQ of NIL-Ep was significantly lower than that of NIL-non Ep under H treatment (Fig. 3A). The texture of rice showed that the hardness was significantly higher in Ep while the viscosity and elasticity were lower (Fig. 3E–G).

We assessed the EQ of the top, middle and bottom panicle parts, and found that the EQ of the middle to the bottom part of NIL-Ep was significantly lower than in NIL-non Ep, which was the key factor affecting the overall EQ (Fig. 3B). For RVA characteristics, the middle and bottom locations of NIL-Ep showed significantly lower breakdown, higher final viscosity and setback values than those of NIL-non Ep (Additional file 1: Table S1). Previous studies demonstrated that rice with high palatability had a higher breakdown and a lower final viscosity and setback than low-palatability varieties (Ma et al, 2017). Our results confirmed that the EQ of midde and bottom position grains of NIL-Ep was lower compared with NIL-non Ep.

Starch and protein account for 70–80% and 7–10%, respectively, of the components in rice endosperm, respectively, and are considered to be the main factors that affecting EQ (Chen et al. 2021). Therefore, the amylose content and protein components of grains in different panicle locations under H treatment were measured to analyze the key factors causing the lower eating quality. There was no significant difference in amylose content among different panicle positions, but there was a significant difference in nitrogen content of middle and bottom grains, which was significantly higher in NIL-Ep than that of NIL-non Ep (Fig. 3C, D).

We tested the protein content in 24 panicle positions, and the results were showed that, under H treatment, the grain protein content in the middle and bottom parts of NIL-EP was significantly higher than the top part, while there was no significant difference among panicle positions of NIL-non Ep (Fig. 3 H, I). Subsequent analysis of the protein components showed that the difference in nitrogen accumulation was the result of the significantly higher prolamin and glutelin contents (Table 2).

**Table 2:** Comparison of protein content traits for NIL-Ep and NIL-non Ep in different positions of panicle

Locus	Panicle type	Accumulation amount (mg grain^−1^)				Relative content (%)				
		ALB	GLO	PRO	GLU	ALB	GLO	PRO	GLU	Total protein
TOP	NIL-Ep	0.196 ± 0.010	0.225 ± 0.009	0.099 ± 0.003	1.440 ± 0.101	0.75 ± 0.04	0.85 ± 0.04	0.38 ± 0.02	5.48 ± 0.27	7.45 ± 0.37
	NIL-non Ep	0.193 ± 0.015	0.227 ± 0.011	0.105 ± 0.005	1.453 ± 0.073	0.71 ± 0.02	0.83 ± 0.02	0.38 ± 0.01	5.30 ± 0.16	7.22 ± 0.22
	p	n.s	n.s	n.s	n.s	*	n.s	n.s	n.s	n.s
MID	NIL-Ep	0.182 ± 0.013	0.214 ± 0.017	0.154 ± 0.005	1.935 ± 0.116	0.70 ± 0.03	0.81 ± 0.04	0.59 ± 0.03	7.64 ± 0.38	9.75 ± 0.69
	NIL-non Ep	0.184 ± 0.009	0.219 ± 0.011	0.092 ± 0.006	1.426 ± 0.057	0.68 ± 0.02	0.80 ± 0.02	0.34 ± 0.01	5.29 ± 0.16	7.11 ± 0.21
	p	n.s	n.s	**	**	n.s	n.s	***	***	**
BOT	NIL-Ep	0.184 ± 0.015	0.219 ± 0.018	0.130 ± 0.010	1.677 ± 0.134	0.72 ± 0.06	0.83 ± 0.07	0.51 ± 0.04	6.61 ± 0.53	8.68 ± 0.49
	NIL-non Ep	0.197 ± 0.010	0.228 ± 0.011	0.105 ± 0.005	1.410 ± 0.070	0.72 ± 0.04	0.83 ± 0.04	0.39 ± 0.02	5.18 ± 0.26	7.12 ± 0.36
	p	n.s	n.s	**	**	n.s	n.s	**	**	**

ALB, Albumin; GLO,Globulin; PRO, Prolamin; GLU, Glutelin

*, **, ***Significance at *p* < .05; *p* < .01; and *p* < .001, respectively

### Nitrogen-Use Efficiency and Grain Protein Accumulation

Previous studies have shown that *dep1* is considered the major gene controlling nitrogen-use efficiency (NUE). In the two years of repeated experiments in 2019 and 2020 (Additional file 2: Table S2), under the high nitrogen condition, the yield of NIL-Ep was 30.6% and 50.4% higher than that of NIL-non Ep. In H treatment, the nitrogen recovery efficiency and physiological NUE were significant higher in Ep than non Ep. There was no significant difference in the yield and NUE under L treatment.

**Fig. 4:**
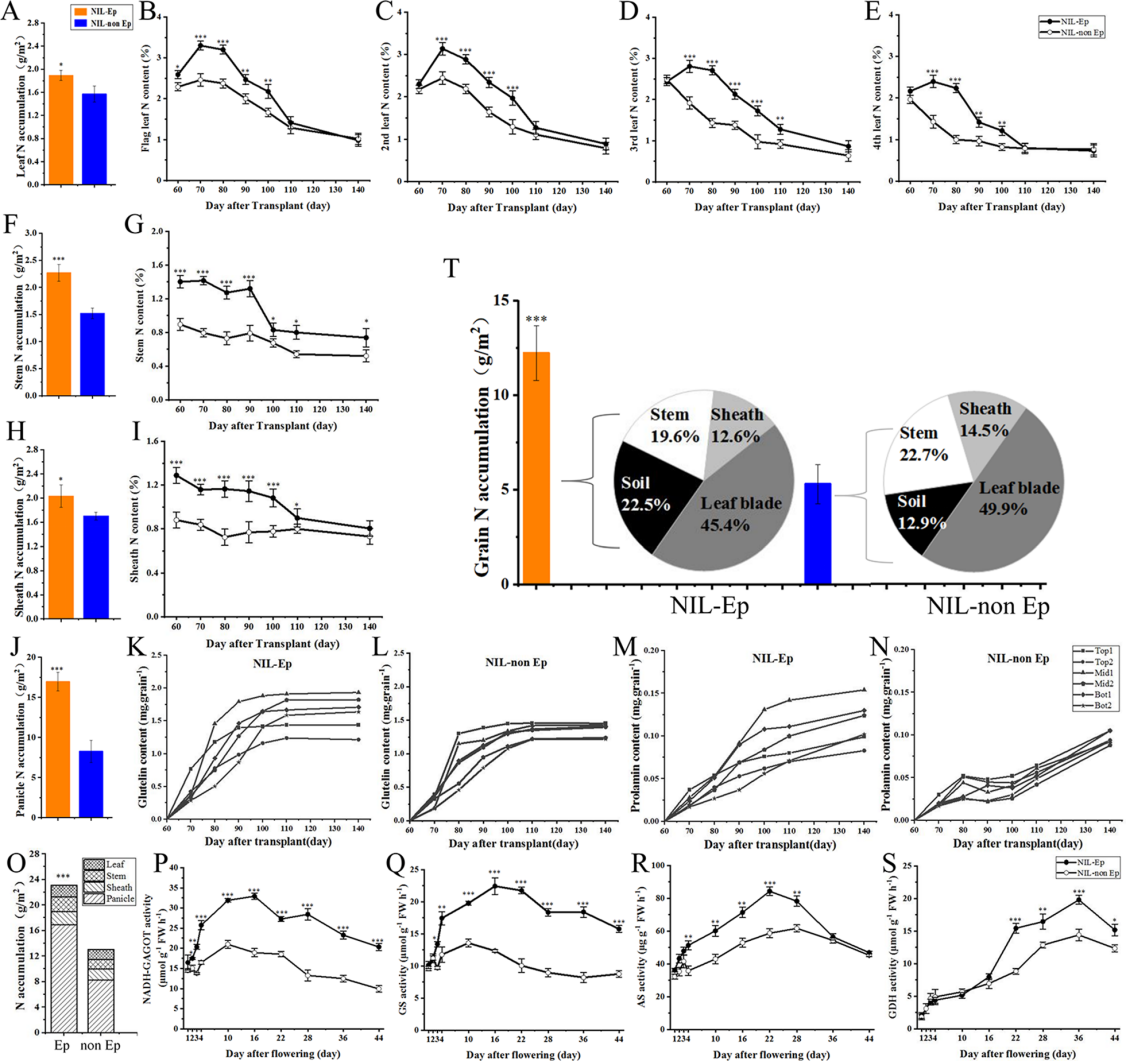
Differences in nitrogen transfer and nitrogen metabolism-related enzyme activities of NIL-Ep and NIL-non Ep plants under high nitrogen treatment. **A** Leaf nitrogen accumulation at maturity stage. **B**–**E** Dynamics of leaf (from flag leaf to 4th leaf) nitrogen content. **F** Stem nitrogen accumulation at maturity stage. **G** Dynamics of stem nitrogen content. **H** Sheath nitrogen accumulation at maturity stage. **I** Dynamics of leaf sheath nitrogen content. **J** Grain nitrogen accumulation at maturity stage. **K**–**N** Dynamics of glutelin and prolamint content in different panicle positions. **O** Total nitrogen accumulation at maturity stage. **P**–**S** Activities of enzymes related to nitrogen metabolism. GS: glutamine synthetase, GOGAT: glutamate synthase, AS: asparagine synthetase, GDH: glutamate dehydrogenase. **T** Origin of nitrogen in panicles from various organs and soils in rice from heading to mature period. The N represent nitrogen element. The *, ** and *** Significance at 0.05, 0.01, and 0.001 level respectively

We examined the nitrogen transport in different organs at harvest stage under the condition of high nitrogen. Nitrogen accumulation of NIL-Ep was significantly higher than that of NIL-non Ep in all organs (Fig. 4A, F, H, J) so that the total nitrogen accumulation of NIL-Ep was significantly higher at harvest (Fig. 4O). From booting to full heading stage, the nitrogen content in leaves still maintained an upward trend in NIL-Ep, while in NIL-non Ep, this phenomenon only occurred in flag leaves and second leaves. In addition, it is interesting that 80–100 d after transplanting, the leaf nitrogen content of NIL-Ep showed a sharp downward trend (Fig. 4B–E), and the same trend also appeared in the stem and sheath organs (Fig. 4G, I). We further analyzed the dynamic changes of the glutelin and prolamin contents of the two genotypes during the grain filling stage, and the found there were marked significant differences in the contents of the two protein components between different panicle locations in NIL-Ep while there was little difference among different locations in NIL-non Ep (Fig. 4K–N).

Nitrogen metabolism processes involves a series of reactions, including inter-conversion of inorganic nitrogen and protein biosynthesis; these processes are highly regulated by both genetic and environmental factors. The enzymes involved in to catalyzing these reactions include glutamine synthetase (GS), glutamate synthase (GOGAT), asparagine synthetase (AS), and glutamate dehydrogenase (GDH), all of which play key roles in the regulation of nitrogen metabolism. We measured the activities of these enzymes in the two genotypes during grain filling. As shown in Fig. 4P–S, significant differences were detected between the two genotypes in GS and NADH-GAGOT activity throughout the grain filling stages. The activity of AS and GDH increased significantly during the periods 4–28 d and 22–44 d after flowering respectively. To clarify the differences in grain nitrogen accumulation derived from different organs and exogenous nitrogen from full heading to harvest stage, we calculated the nitrogen accumulation in grains of NILs and the proportions contribution by each plant part. The total nitrogen accumulation in NIL-Ep grains was significantly higher than that in NIL-non EP grains, and the origin of grains nitrogen derived from various organs suggested that the ability of NIL-Ep to absorb nitrogen was significantly higher than that of NIL-non Ep at the grain-filling stage under the high nitrogen condition (Fig. 4T).

## Discussion

Proposed from ideal plant type breeding, Ep is widely used in super rice breeding, such as Shennong 265, a typical variety that has not only higher yield potential, but also has better performance in lodging resistance and disease resistance; Ep-type rice has therefore replaced Japanese *japonica* rice (e.g. Toyonishiki, Akihikari and Akitakomachi) as the main type of *japonica* rice grown in northern China (Xu et al. 2016a). Previous studies have shown that *dep1* expression is positively regulated by nitrogen fertilizer (Palme et al. 2014). Under high nitrogen conditions (120 kg/hm^2^), Ep varieties show significantly improved plant type and yield-related traits, compared with non Ep varieties. Under low nitrogen conditions (60 kg/hm^2^), the yield of Ep varieties is greatly reduced, and can be even lower than that of the non Ep varieties (Tang et al. 2017b). This means that Ep varieties cannot reach their full production potential given a limited nitrogen supply. Our results led to the same conclusion (Fig. 1A–H, Table 1). In four years of field trials, the parents and NILs showed similar patterns in yield. Under L treatment, Ep showed no obvious yield advantage in yield. However, the yield was significantly increased under H treatment. For non Ep type, there was no significant difference in yield between the two fertilizer treatments. In our analysis of yield components, the increased of grain number in the middle to bottom part had the highest contribution to yield (Fig. 2E, F).

Rice is the main food crop for more than half of the world's population and its eating quality is the main index that determines its value (Zhu et al. 2020). The quality of cooked rice is a complex trait, affected not only affected by the inherent physical characteristics of the kernel, but also by its composition (Vidal et al. 2007). There is a common view that the protein content has a positive correlation with the hardness of cooked rice (Amagliani et al. 2017). The viscosity profiles of rice flour with similar starch properties supported the view that protein content negatively correlated with gelatinization temperature and peak viscosity (Bornhorst et al. 2013; Fitzgerald et al. 2003). Analysis of the formation of protein–starch matrixes showed that protein inhibited starch maximum swelling and restricted its ability to absorb water (Derycke et al. 2005; Saleh. 2017). Our research institution has conducted research on the quality and composition of grains in Ep type rice, but because of the limitations on using genetically modified materials for eating, there is no clear conclusion about the influence of Ep on EQ (Fei et al. 2019). In this study, Ep decreased the EQ through enhancing the protein content of grains in the middle and bottom panicle positions (Fig. 3). The significant increase in prolamin and glutelin content was the main factor leading to increased grain protein content in Ep rice(Table 2).

Nitrogen uptake and use in rice involves multiple physiological and biochemical processes of absorption, transport, assimilation, remobilization and allocation (Chen et al. 2021). The *dep1* gene regulates nitrogen uptake and metabolism by affecting *OsAMT1;1,* which is associated with ammonium uptake (Sun et al. 2014). *dep1* enhances the ability of the root system to absorb ammonia nitrogen, allowing the plants to accumulate more nitrogen accumulation, and then improves the utilization efficiency of nitrogen (Xu et al. 2016b). In addition, *dep1* overexpression lines showed higher expression of *GS* and *GOGAT* genes, and thus higher nitrogen metabolic activity than the wild type under high nitrogen conditions (Zhao et al. 2019). In our research, under H treatment, Ep had higher nitrogen use efficiency than non Ep (Table S2), and nitrogen accumulation in all organs was significantly higher (Fig. 4). During the grain-filling period, Ep stimulated nitrogen absorption and redistribution through enhancing the activity of nitrogen metabolism-related enzymes (Fig. 4P-S). At the same time, the absorption and utilization ratio of exogenous nitrogen in Ep was significantly higher than that in non Ep (Fig. 4T).

**Fig. 5:**
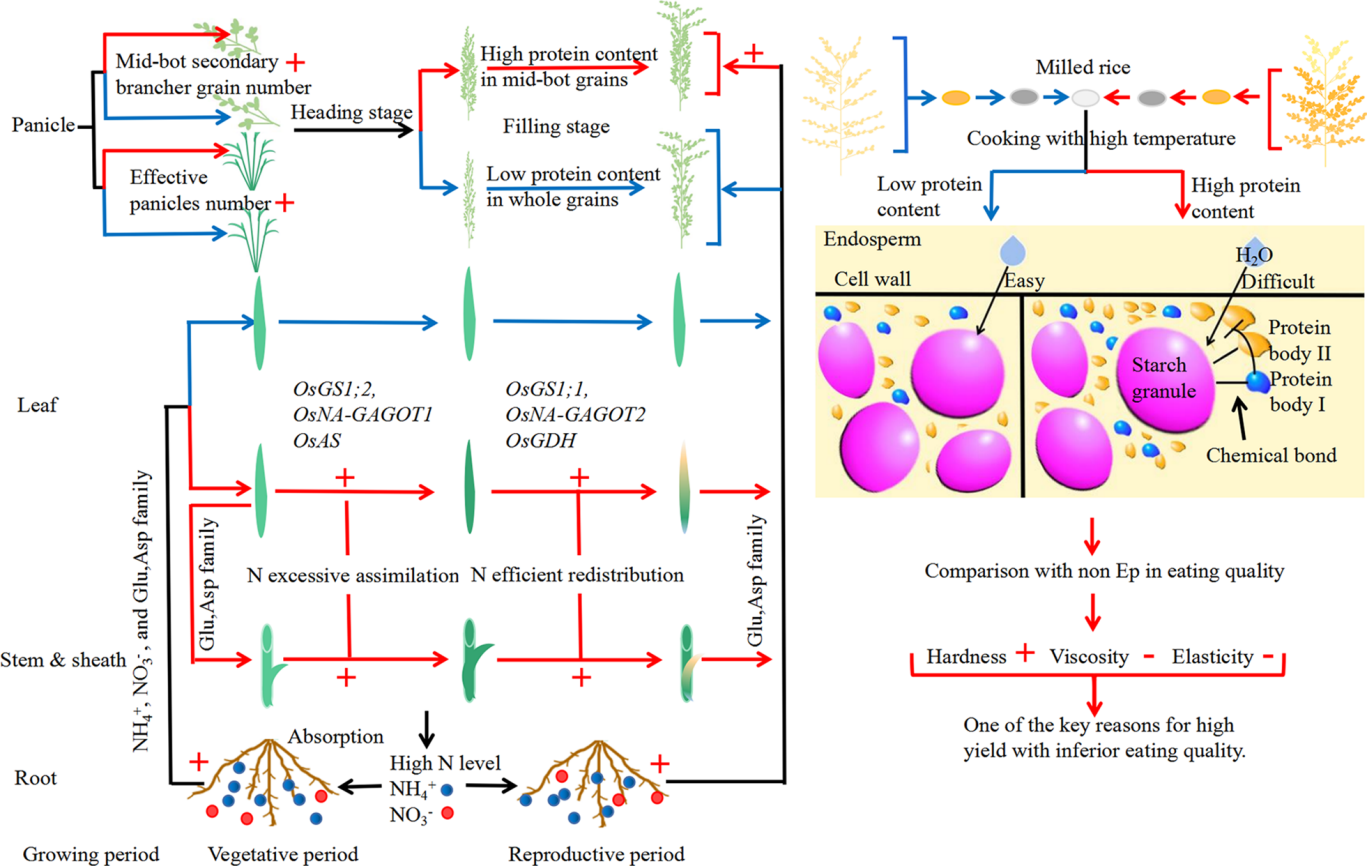
Diagram of Nitrogen utilization of Ep and non Ep panicle type at different growth stages under high nitrogen condition, and interaction between protein and starch granule during cooking. Details are described in the text. Red and blue lines represent the growth and development process of Ep and non Ep type respectively, black lines for common process. Red plus and minus indicate promoting and demoting effect of Ep compared with non Ep type

Researchers have conducted extensive studies on the effect of Ep on yield and nitrogen use efficiency, but there was no clear conclusion on the effect of Ep on EQ. Building on previous studies and the results of this experiment, we propose a simplified model to explain its impact on EQ from the perspective of nitrogen assimilation and redistribution (Fig. 5). Under high nitrogen conditions, at the vegetative stage the Ep type has significantly higher nitrogen accumulation as a result of enhanced nitrogen absorption of roots and the assimilation in leaves, which enables an increase of panicle number and grain number per panicle. When the filling stage begins, nitrogen is remobilized by nitrogen metabolism related enzymes in the leaves and other vegetative parts and accumulates in the grains. Especially in the middle to late stages of grain filling, the rapid transfer of nitrogen content from other organs leads to a significant increase in protein content (mainly glutelin and prolamin protein) in the middle to bottom grains. Grain protein was shown to compete to absorb water and restrict the swelling of starch granules, which in turn affected the texture of the cooked rice (Saleh 2017). The rice kernel is a heterogeneous assemblage of the distinct components (Cai, et al. 2014). In the fully mature rice kernel, the protein bodies (PB) were tightly gathered along the cell walls and surrounded the starch granules, which were held with chemical bonds (Zhu et al. 2020). The steric hindrance effect of the PB and starch–protein interactions were the main factors limiting starch gelatinization. The surrounding protein structure or the bonds between starch and protein, restricted water penetration into the starch granules. This led to a reduction in water absorption and restricted the space available for gelatinization, thereby inhibiting the expansion of starch granules (Fitzgerald et al. 2003). In addition, protein has good thermal stability and hydrophobicity, making it resistant to changing its conformation during cooking. Therefore, compared with the non Ep type, the additional protein content in Ep grains is one of the key reasons for the decrease of rice EQ.

In addition, our study showed that Ep had a positive effect on yield and a negative effect on EQ under high nitrogen input conditions. How to achieve EQ improvement while maintaining high yield has become the aim of many agricultural researchers. As breeding and genetic objectives, we suggest the following. (1) Cultivating the Ep type with higher percentage of grains in top positions to reduce the adverse effect of middle and bottom grains on EQ (Additional file 3: Fig. S1). (2) Introducing dominant genes controlling grain length such as *GS3* (Fan et al. 2006), *GL3.1* (Qi et al. 2012; Zhang et al. 2012), *GLW7* (Si et al. 2016) and *GS2* (Che et al. 2015; Hu et al. 2015) aiming to increase yield by improving grain weight rather than increasing the number of weak grains. (3) Mining additional variations to increase the Ep haplotypes available for breeding; meanwhile creating different type of *dep1* alleles based on gene editing,technology that learn from the way for improving *Wx* and *SD1* gene did (Huang et al. 2021; Biswas et al, 2020). (4) To reduce or delay the excessive transfer of nitrogen during grain filling, gene editing technology might be used to reduce the expression of genes controlling nitrogen metabolism-related enzyme activities (such as *OsGS*,*OsNA-GOGOT*,*OsGDH*) and fine-tune grain protein content in rice. From the cultivation point of view: according to the characteristics of different varieties, we suggest reasonable regulation of nitrogen fertilizer application to explore the balance of yield and quality, aiming to maintain high yield while also achieving quality improvement. These will be the key research directions that need to be further studied and clarified in future work, and will also provide an important theoretical basis for the realization of EQ improvement alongside super-high yield.

## Conclusions

Under high nitrogen conditions, Ep significantly increased yield by increasing the effective panicle number and the number of grains in the middle and lower part of the panicle, but it was additional grains that led to the decrease of the overall EQ. The protein content of these grains (mainly prolamin and glutelin) was significantly higher, which reduced the characteristic values of EQ and thus diminished the taste. At the same time, Ep type rice showed high nitrogen use efficiency. At the filling stage, the key enzyme activities of nitrogen metabolism in Ep flag leaves were significantly higher than in non Ep, which promoted nitrogen reassimilation. This in turen enabled the rapid increase of protein content in the grains, which was one of the key factors leading to reduced grain EQ.

## Materials and Methods

### Plant Material and Experimental Site

We constructed a pair of rice (*Oryza sativa L*.) NIL lines denoted as NIL-Ep, NIL-non Ep, in the LG5 and AKI backgrounds (Fig. 1A). To further determine the genome composition of the two NIL lines, we performed high-throughput sequencing analysis on both. First, the raw paired-end sequence data for each sample were generated by Illumina HiSeq4000 sequencing. Then, raw fastq files were filtered by using fastp software with default settings. Clean reads were mapped to the rice reference genome Nipponbare-IRGSP v1.0 through using BWA software. The sequence variants between NIL-Ep and NIL-non Ep were detected by Samtools and GATK4 software for joint genotyping. Then 504,474 SNPs and 88,169 indels in genome-wide were identified after filtering using vcftools software according to the thresholds: –min-alleles 2—max-alleles 2—max-missing 1—minDP 3—minQ 30. Finally 2048 SNPs and 1322 indels were obtained by considering only the polymorphism between NIL-Ep and NIL-non Ep; thus the genetic identity of the two materials was 99.43%. The distribution of these 3370 variants was visualized as Additional file 4: Fig. S2, in which the genomic region of *dep1* exhibit strong genetic differentiation.

The experiment was carried out at the farm of Shenyang Agricultural University, Shenyang, China (41.8° N; 123.4° E), during the rice growing seasons in 2018 to 2021. Germinated seeds were grown in the paddy field, and seedlings raised in the field with the sowing date of April 24 were transplanted on May 24 at a spacing of 0.30 m between rows and 0.15 m between plants, with one seedling per hill. The materials were arranged in a randomized block design with three replicates, and each replicate block contained at least 800 plants. The design used three nitrogen treatments, respectively 0 kg/hm^2^ nitrogen (control check, CK) area, 11.25 kg/hm^2^ nitrogen (low, L; as used in high quality cultivation in Japan) and 22.5 kg/hm^2^ nitrogen (high H; as used in high yield cultivation in northern China). Input of P and K and production management were the same as conventional production methods. Treatments and varieties used for experiments in pots were the same as the field, with two plants per pot.

### Evaluation of Yield and Yield Components

At the heading stage, plant height (PH, in cm) and heading date were recorded when 50% of the plants showed emerged panicles. At maturity, plants from a 4-m^2^ area in each plot were harvested for grain yield (GY) measured at 14% moisture content after being air-dried. Panicles were selected from six main stems per hill, replicated three times, giving 18 plants sampled for trait evaluation, comprising panicle number per plant (PN), panicle length (PL), panicle number per plant (PNP), grain number per panicle (GNP), filled grain number per panicle (FGN), primary grain number (PGN), secondary grain number (SGN), and thousand-grain weight (TGW).

### Nitrogen Element Content and Nitrogen Use Efficiency

The nitrogen element content of the samples was analyzed with a vario MACRO cube (Elementar Co., Hanau, Germany), which is based on the Dumas combustion method. The operation and parameter setting were according to Tang et al. (2019). The agronomic nitrogen use efficiency and apparent nitrogen recovery efficiency were calculated according to Chen et al. (2018).

### Nitrogen Accumulation Amount and Component Contributions

The following parameters were calculated: for each plant part, Nitrogen accumulation amount (NA) = W_dry weight_ × Nitrogen element content; leaf contribution rate (LCR) = (NA_full heading stage leaf_ − NA_maturity stage leaf_)/(NA_maturity stage panicle_ − NA_full heading stage panicle_) × 100%; stem contribution rate (SCR) = (NA_full heading stage stem_ − NA_maturity stage stem_)/(NA_maturity stage panicle_ − NA _full heading stage panicle_) × 100%; Sheath contribution rate (SHCR) = (NA_full heading stage sheath_ − NA _maturity stage sheath_)/(NA_maturity stage panicle_ − NA _full heading stage panicle_) × 100%; soil contribution rate = 100% − LCR − SCR − SHCR.

### Eating Quality Evaluation

Assays of taste and palatability in cooked rice were conducted on an STA1A rice taste analyzer (STA1A; Satake) using the method of Champagne et al. (1996) with minor modification. The taste sensory evaluation panel was made up of 30–35 people of different genders, different ages and with professional ability to identify EQ. Eight samples (including one control sample) were assessed each time. A control sample was set for sensory evaluation to better distinguish more reliably the taste and other sensory among varieties. On the basis of the hardness, viscosity, elasticity, appearance, taste, palatability and cold rice texture of the sample (boiled rice), each taster gave an overall score after comparing with the control sample; the maximum score was 100 points. The average value was calculated from scoring result of each evaluator, to give the overall result for EQ of the sample. The calculated result was expressed to two decimal places.

### Amylose Content and Rapid Visco Analyser (RVA) Determination

Apparent amylose content (AAC, %) was determined from the colorimetric reaction of the amyloseiodine complex developed using the method of ISO 6647 (International Organization for Standardization). The absorbance of the test was measured at a wavelength of 620 nm was measured against the blank solution using a spectrophotometer (Lambda 365; Perkin Elmer). AAC% was calculated using a standard curve made from four rice samples with known AAC% (1.5%, 9.2%, 17.1% and 26%). Rice pasting properties were measured using a Rapid Visco Analyser (RVA) (TechMaster RVA; Perten) using samples of milled rice flour according to the method of Umemoto et al. (2004). The peak time, pasting temperature, peak viscosity, trough viscosity, final viscosity and their derivative parameters, breakdown and setback, were recorded using Thermocline for Windows software (version 1.2). Using the standard method of the American Cereal Chemistry Association Operating Regulations (1995-61-02), the water content of rice flour was 12%, the sample volume was 3 g, and the distilled water volume was 25 ml.

### Measurement of Total Protein and Protein Components in Grains

Total protein content was determined by Kjeldahl method. The content of protein components was determined according to the methods of Ju et al. (2001). A sequential extraction method was used to extract and separate different components using distilled water, 5% NaCl, 70% ethanol and 0.1 mol/L NaOH in turn. The protein content of each component was determined by the Coomassie brilliant blue method.

### Enzyme Activity Determination

The activity of GS was determined according to Sun et al. (2014), NADH-GOGAT according to Singh and Srivastava (1986), GDH according to Yamaya et al. (1984), and AS was conducted according to Nakano et al. (2000).

### Statistical Analysis

The data were statistically analyzed with Excel 2003 (Microsoft Office 2003) and SPSS 24.0 for Windows (IBM Corporation), and means were tested by least significant difference at *P* < 0.05 (LSD 0.05).

## Supplementary Information

**Additional file 1: Table S1**. Comparison of RVA traits for NIL-Ep and NIL-non Ep in the Akitakomachi and Liaogeng5 backgrounds.

**Additional file 2: Table S2**. Comparison of N use efficeiency for NIL-Ep and NIL-non Ep.

**Additional file 3: Figure S1**. The schematic diagram of comparison between current panicle type and breeding target panicle type.

**Additional file 4: Figure S2**. The distribution of 3,370 genome-wide variants between NIL-Ep and NIL-non Ep, color gradation indicated the variants number with in 1Mb window size. The arrow indicates the physical position of the DEP1 gene.

## Data Availability

All data supporting the conclusions of this article are provided within the article (and in the Additional files).

## Abbreviations

AKI: Akihikari
ALB: Albumin
AS: Asparagine synthetase
CK: Control check
dep1: Dense and erect panicle 1
Ep: Erect panicle
EQ: Eating quality
FGN: Filled grain number per panicle
GAGOT: Glutamine- 2-oxoglutarate aminotransferase
GDH: Glutamate dehydrogenase
GLO: Globulin
GLU: Glutelin
GNP: Grain number per panicle
GS: Glutamine synthetase
H: High nitrogen
HD: Heading date
L: Low nitrogen
LG5: Liaogeng 5
N: Nitrogen
NA/NADH: Nicotinamide adenine dinucleotide
NILs: Near-isogenic lines
NUE: Nitrogen use efficiency
PBI: Protein body I
PBII: Protein body II
PBN: Primary branches
PGN: Primary grain
PH: Plant height
PL: Panicle length
PNP: Panicle number per square meter
PRO: Prolamin
RVA: Rapid visco analyser
