# Correction to: Dissecting of the Deterioration in Eating Quality for Erect Panicle (Ep) Type High Yield *Japonica* Super Rice in Northest China

**DOI:** 10.1186/s12284-022-00565-5

**Published:** 2022-03-30

**Authors:** Sibo Chen, Shuangjie Chen, Yihui Jiang, Qing Lu, Zhongyuan Liu, Wanying Liu, Xuhong Wang, Wenhua Shi, Quan Xu, Jian Sun, Fan Zhang, Liang Tang

**Affiliations:** 1 Rice Research Institute, Shenyang Agricultural University/Key Laboratory of Northern Japonica Super Rice Breeding, Ministry of Education, Shenyang, 110866 China; 2 Institute of Crop Sciences/National Key Facility for Crop Gene Resources and Genetic Improvement, Chinese Academy of Agricultural Sciences, 12 South Zhong-Guan-Cun Street, Haidian District, Beijing, 100081 China

## Correction to: Rice (2022) 15:15. 10.1186/s12284-022-00561-9

Unfortunately, in the original publication of the article, the Figs. 1, 2, 3 and 4 and Fig. S1 (Additional file 3) were published without part labels. The corrected figures are given in this Correction article. The original article has been corrected.

**Fig. 1:**
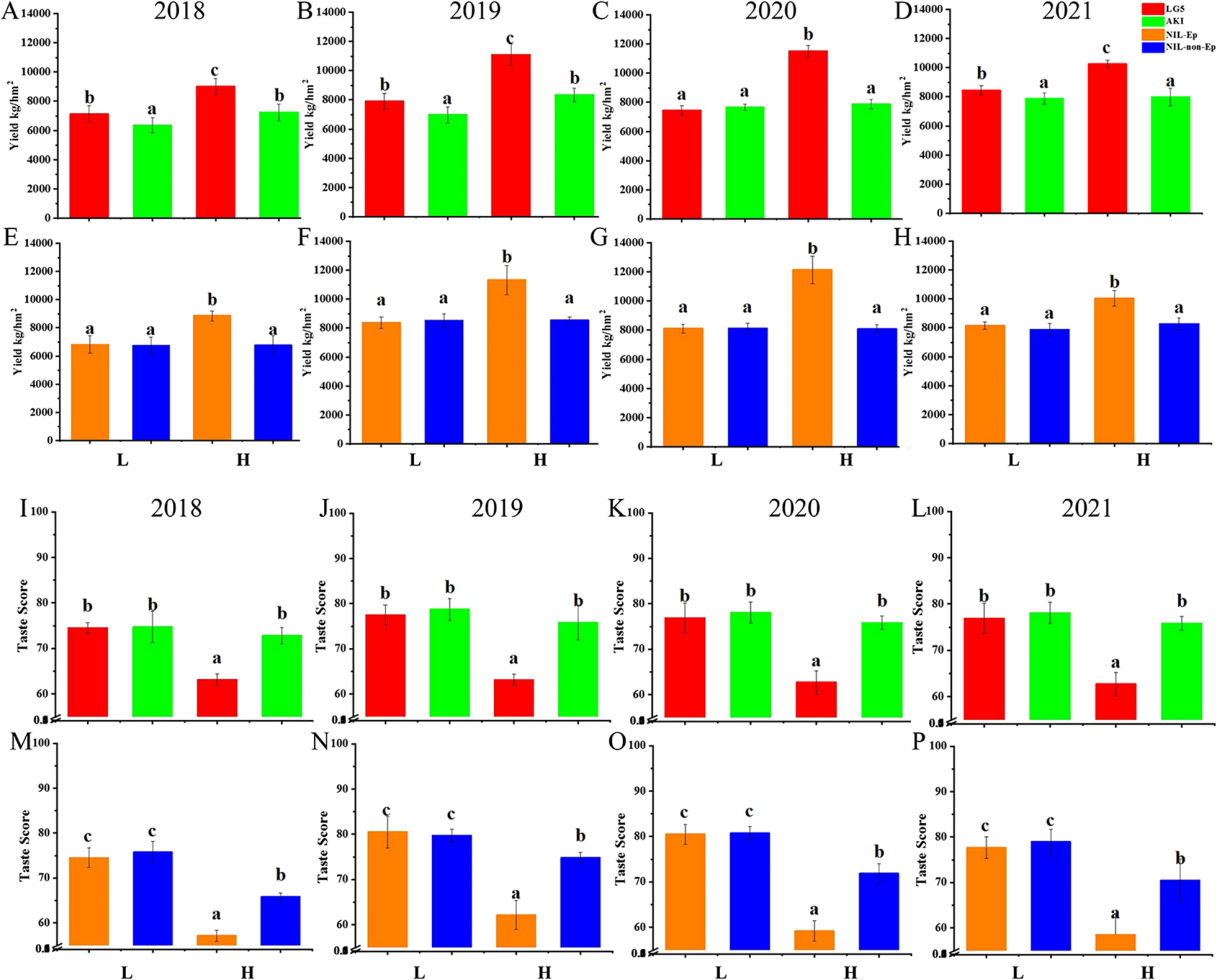
Phenotypes of yield and taste quality of test meterials from 2018 to 2021. **A**–**H** Yield for LG5, AKI, NIL-Ep and NIL-non Ep. **I**–**P** Taste score for LG5, AKI, NIL-Ep and NIL-non Ep. (a), (b) Significance at the 0.05 level. **L**, **H** Low and high nitrogen treatments respectively

**Fig. 2:**
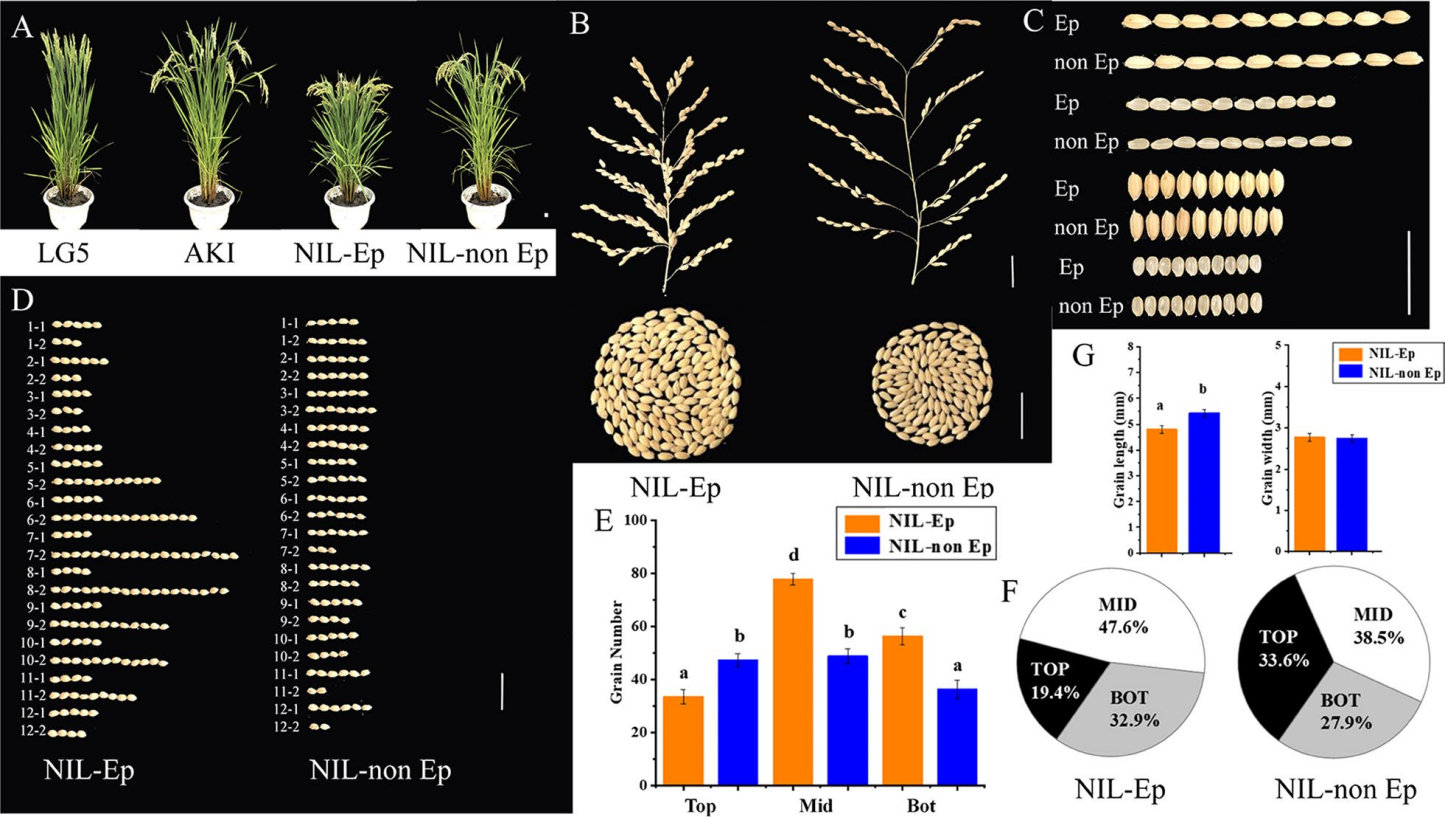
The yield performance and grain shape of NIL-Ep and NIL-non Ep plants. **A** The four experimental materials plant in pot under high nitrogen treatment. **B** The panicles and grain numbers per panicle of the NIL-Ep and NIL non Ep. **C** The grain size of the NIL-Ep and NIL non Ep. **D** The grain numbers of different panicle locations for NIL-Ep and NIL non Ep, divided rice panicles into 24 positions from 1–1 to 12–2 according to the origin positions of branches. e.g. 1–1 represented primary branches at the top and 12–2 represented the secondary branches at the bottom. **E** Difference analysis of grain number in different panicle parts, the panicle is divided into 3 parts namely top (top, from 1–1 to 4–2 panicle positons), middle (mid, from 5–1 to 8–2 panicle positons) and bottom (bot, from 9–1 to 12–2 panicle positons) respectively. **F** Proportion of grain number in different panicle positions. **G** Difference analysis of grain shape. (a), (b) Significance at the 0.05 level. Scale bar, 2 cm

**Fig. 3:**
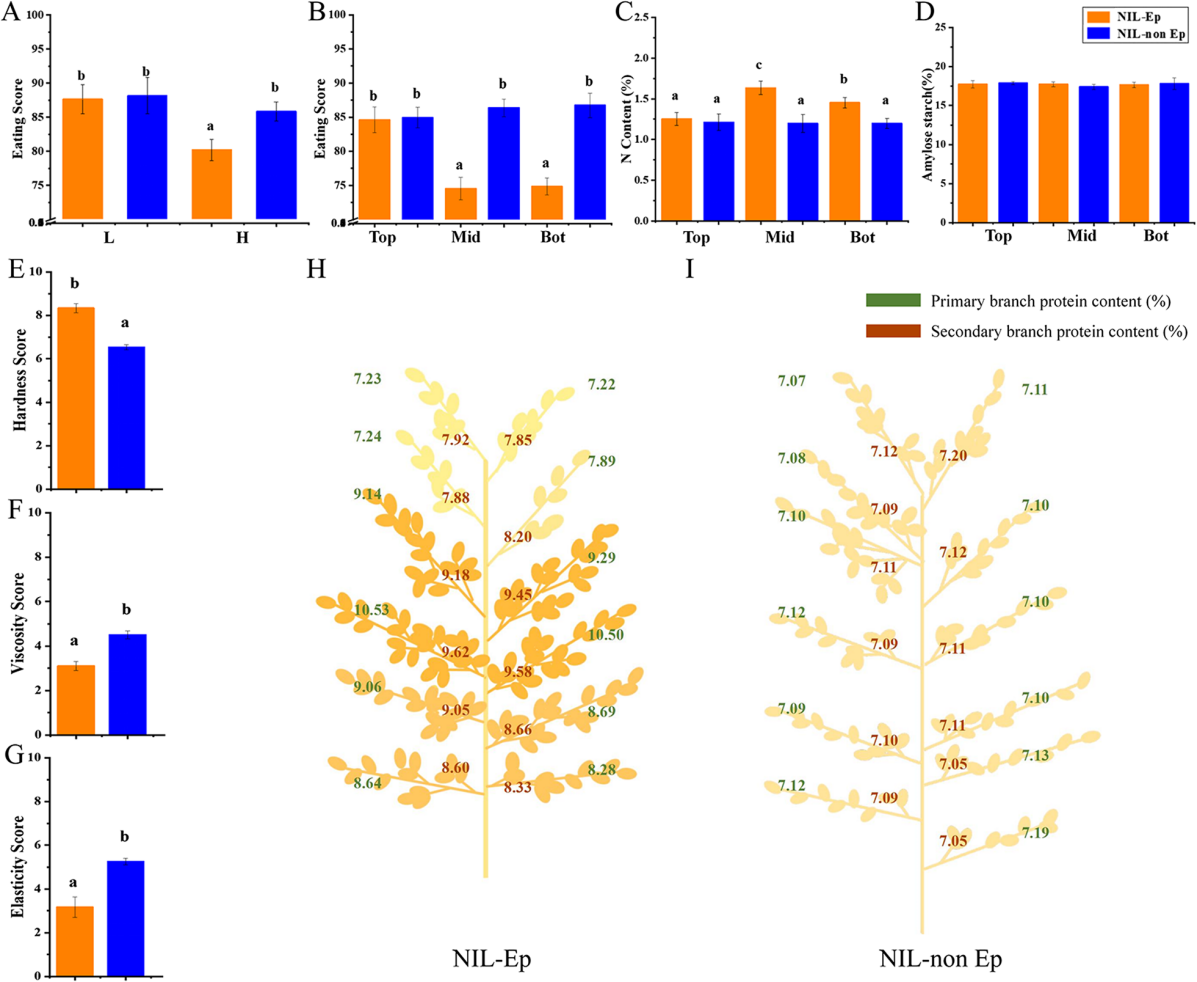
The eating quality performance and grain protein, starch content of NIL-Ep and NIL-non Ep plants. **A** Eating quality under two nitrogen fertilizer treatments. **L**, **H** Low and high nitrogen treatments respectively. **B** Eating quality of different panicle locations under H treatment, the panicle is divided into 3 parts namely top (top, from 1–1 to 4–2 panicle positons), middle (mid, from 5–1 to 8–2 panicle positons) and bottom (bot, from 9–1 to 12–2 panicle positons) respectively. C N content of different panicle locations under high nitrogen treatment. **D** Amylose content of different panicle locations under high nitrogen treatment. **E**–**G** Components of eating quality of NIL-Ep and NIL-non Ep under high nitrogen treatment. **H**, **I** Protein content (%) of different panicle locations under high nitrogen treatment. (a), (b) Significance at the 0.05 level

**Fig. 4:**
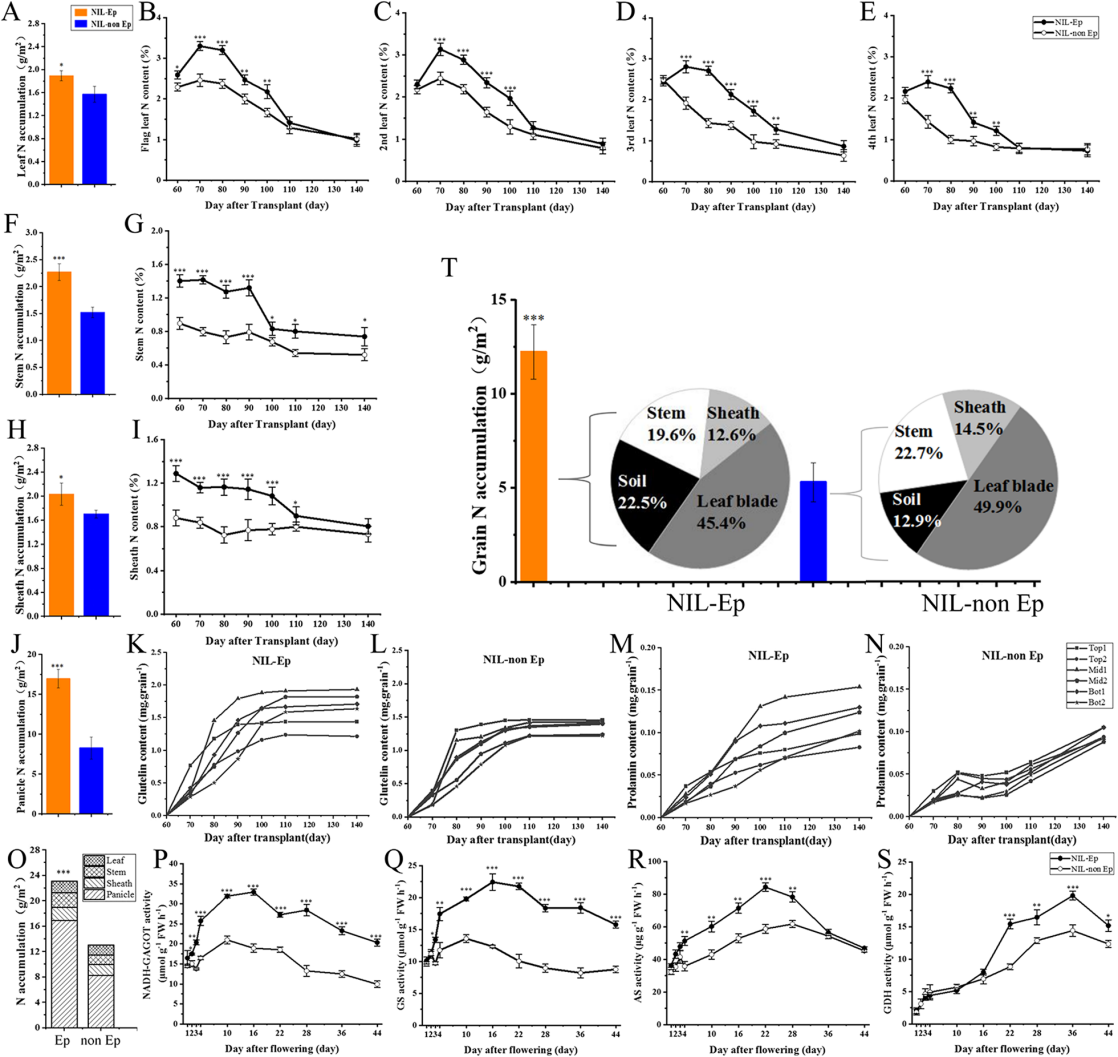
Differences in nitrogen transfer and nitrogen metabolism-related enzyme activities of NIL-Ep and NIL-non Ep plants under high nitrogen treatment. **A** Leaf nitrogen accumulation at maturity stage. **B**–**E** Dynamics of leaf (from flag leaf to 4th leaf) nitrogen content. **F** Stem nitrogen accumulation at maturity stage. **G** Dynamics of stem nitrogen content. **H** Sheath nitrogen accumulation at maturity stage. **I** Dynamics of leaf sheath nitrogen content. J Grain nitrogen accumulation at maturity stage. **K**–**N** Dynamics of glutelin and prolamint content in different panicle positions. **O** Total nitrogen accumulation at maturity stage. **P**–**S** Activities of enzymes related to nitrogen metabolism. GS: glutamine synthetase, GOGAT: glutamate synthase, AS: asparagine synthetase, GDH: glutamate dehydrogenase. **T** Origin of nitrogen in panicles from various organs and soils in rice from heading to mature period. The N represent nitrogen element. The *, ** and *** Significance at 0.05, 0.01, and 0.001 level respectively

## Supplementary Information

**Additional file 3**: **Figure S1.** The schematic diagram of comparison between current panicle type and breeding target panicle type.

