# Supplementary material for: Dissecting of the Deterioration in Eating Quality for Erect Panicle (Ep) Type High Yield Japonica Super Rice in Northest China
Source: Rice (N Y). 2022 Mar 8;15:15. doi: 10.1186/s12284-022-00561-9 (PMC8901826; doi:10.1186/s12284-022-00561-9)
Supplement: Supplementary file 3 — Additional file 3: Figure S1. The schematic diagram of comparison between current panicle type and breeding target panicle type. [file 12284_2022_561_MOESM3_ESM.doc]

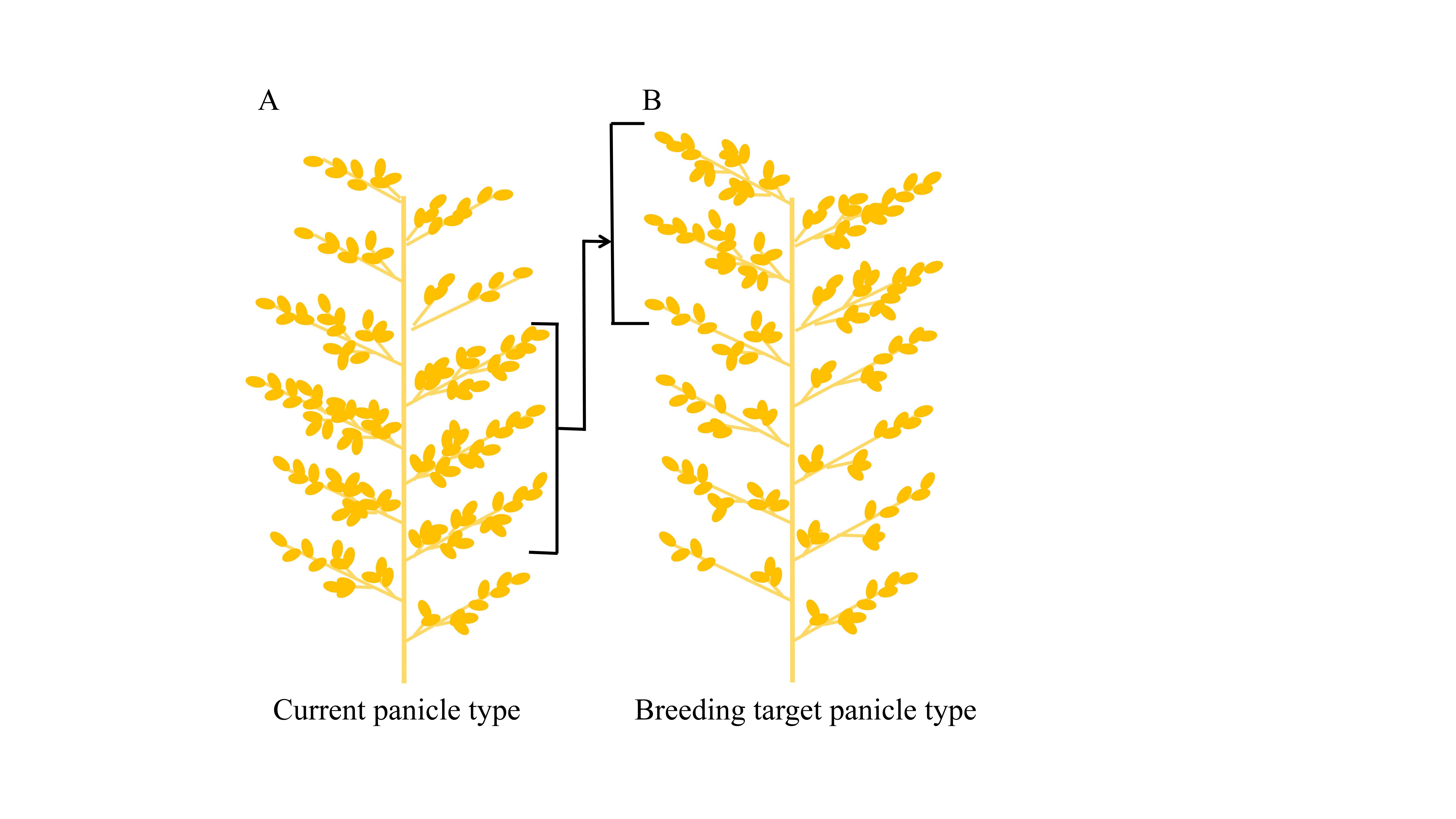


**Additional file 3: Figure S1.** The schematic diagram of comparison between current panicle type and breeding target panicle type.
