# Supplementary material for: Dissecting of the Deterioration in Eating Quality for Erect Panicle (Ep) Type High Yield Japonica Super Rice in Northest China
Source: Rice (N Y). 2022 Mar 8;15:15. doi: 10.1186/s12284-022-00561-9 (PMC8901826; doi:10.1186/s12284-022-00561-9)
Supplement: Supplementary file 4 — Additional file 4: Figure S2. The distribution of 3,370 genome-wide variants between NIL-Ep and NIL-non Ep, color gradation indicated the variants number with in 1Mb window size. The arrow indicates the physical position of the DEP1 gene. [file 12284_2022_561_MOESM4_ESM.doc]

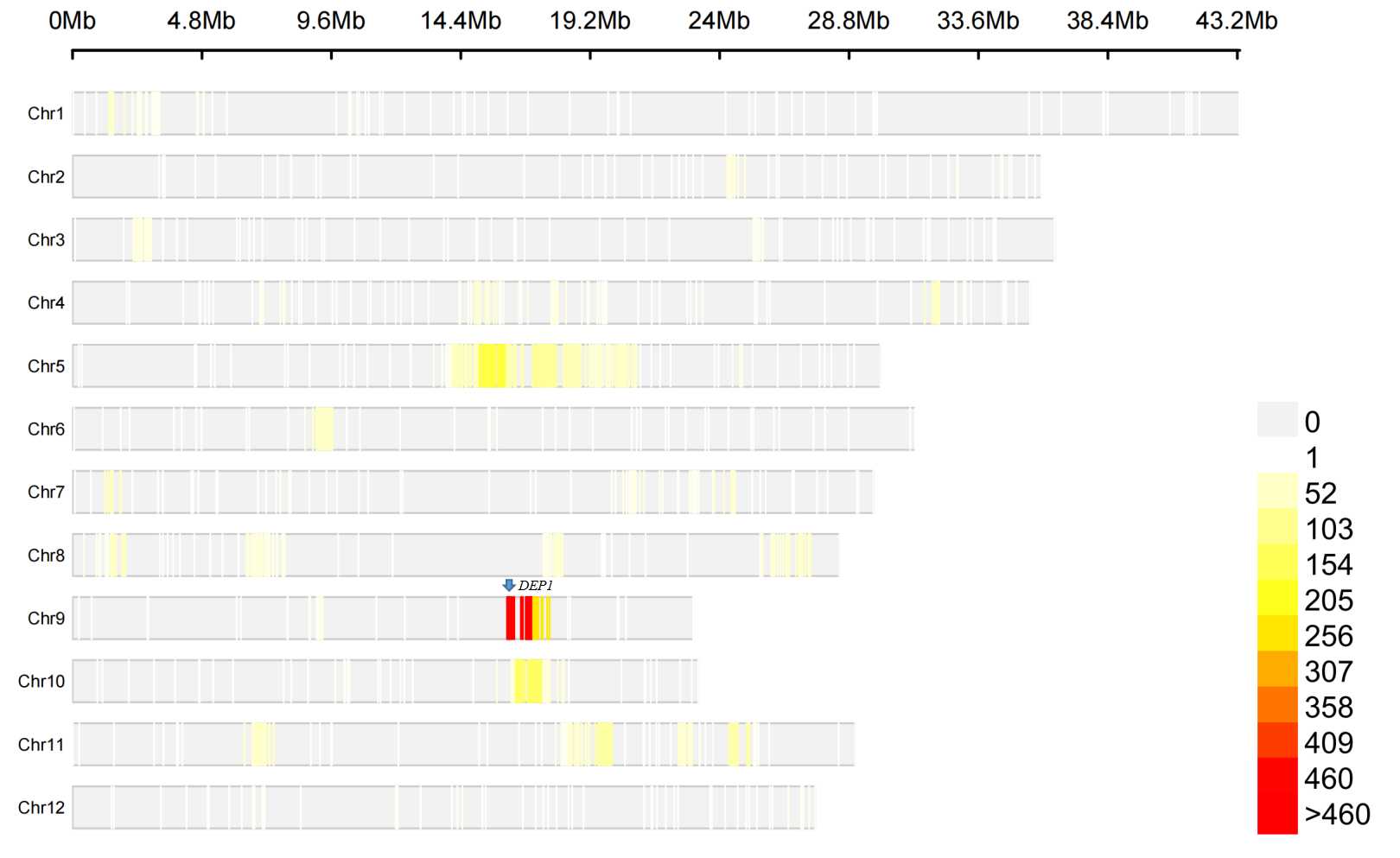


**Additional file 4: Figure S2.** The distribution of 3,370 genome-wide variants between NIL-Ep and NIL-non Ep, color gradation indicated the variants number with in 1Mb window size. The arrow indicates the physical position of the *DEP1* gene.
